# Regulation of Oil Penetration, Lipid Oxidation, and Flavor Characteristics in Batter-Coated Fried Fish Cubes: The Functional Implications of Hydrocolloids

**DOI:** 10.3390/gels11100781

**Published:** 2025-09-30

**Authors:** Yujie Li, Yueqi Wang, Yanyan Wu, Hengli Bai, Yongqiang Zhao, Huan Xiang, Jun Li

**Affiliations:** 1 College of Light Industry and Food, Zhongkai University of Agriculture and Engineering, Guangzhou 510225, China; liyujie0404@163.com; 2 South China Sea Fisheries Research Institute, Chinese Academy of Fishery Sciences, Guangzhou 510300, China; wangyueqi@scsfri.ac.cn (Y.W.); wuyygd@163.com (Y.W.); henglibai55@163.com (H.B.); zhaoyq@scsfri.ac.cn (Y.Z.); skyxianghuan@163.com (H.X.)

**Keywords:** hydrocolloids, battered frying, lipid oxidation, free fatty acid, flavor

## Abstract

This study elucidates the mechanisms through which hydrocolloids inhibit oil penetration and improve the sensory quality of batter-coated fried fish cubes. Specifically, guar gum (GuG), linseed gum (LG), acacia senegal gum (AS), and gellan gum (GeG) were individually incorporated into the batter coating system at an addition level of 0.1%. The results indicated that the 0.1% LG-supplemented group significantly increased batter viscosity by 74.9% compared to the control, which in turn improved batter pickup by 26.1% and frying yield by 8.1%. Rheological analysis revealed that hydrocolloid-incorporated batters exhibited markedly higher storage modulus and loss modulus compared to the control group, with a lower loss tangent. Experimental results indicated that hydrocolloids effectively reduced oil absorption and mitigated the rate of lipid oxidation in fried fish cubes while promoting the release of key flavor compounds. Notably, fried fish cubes coated with GuG, when fried at 170 °C, not only reduced oil absorption but also facilitated the formation of critical flavor compounds. These findings provide a theoretical foundation for optimizing fried food processing and flavor control.

## 1. Introduction

Fried foods, a common category of thermally processed products, encompass a diverse range of types, including fried chicken, pork chops, fried vegetables, and battered fish cubes. Consumers widely favor them for their golden-appearing color, crispy exterior, and tender interior texture [1]. During high-temperature frying, the rapid vaporization of internal moisture creates abundant porous structures in foods, forming channels for oil penetration and leading to a significant increase in the oil content of fried products [2]. This phenomenon is supported by the study on fried potato chips: the number of pores with a diameter of 10–200 μm increased significantly, and this change led to an increase in the oil content of the potato chips to 61.20% at 160 s of frying [3]. Studies indicate that the oil content of certain fried foods can reach 50% of their weight; long-term excessive consumption of such high-fat products significantly elevates the risk of obesity, cardiovascular diseases, and hypertension [4]. Moreover, inappropriate frying conditions such as excessively high temperatures, prolonged frying durations, or repeated use of the same oil often trigger excessive lipid oxidation, hydrolysis, and polymerization, leading to the formation of harmful substances such as oxides and heterocyclic amines, which pose potential health risks [5]. Exploring technical methods to balance oil absorption control and sensory quality optimization is crucial for meeting consumers’ demands for healthy and delicious foods.

To reduce oil absorption, various pretreatment technologies have been developed and applied in the field of fried food processing, primarily including surface coating, pre-freezing, pre-microwave heating, hot air convection drying, and osmotic dehydration. These methods have been validated to effectively reduce oil uptake in fried products [6]. For instance, Dehghannya’s study on potato samples showed that applying an ultrasound pretreatment (28 kHz, 15 min) prior to frying resulted in a 14% reduction in oil uptake [7]. Among them, incorporating functional additives (especially hydrocolloids) into surface coatings has shown remarkable efficacy in lowering the oil penetration of fried foods [8]. Jouki et al. [9] found that applying an active batter coating containing papaya seed gum and carvacrol microcapsules reduced the oil uptake of fried chicken nugget samples by 33.21%. Hydrocolloids, as a class of long-chain polymers containing numerous hydroxyl groups, exhibit exceptional hydration capacity. Under heating conditions, they form a stable gel network that not only minimizes moisture loss during frying but also acts as a physical barrier to inhibit oil penetration into the food matrix. Among these, polysaccharide-based materials, such as cellulose derivatives, plant mucilages, and gums, have been widely applied in food coating technologies as typical hydrocolloids [10]. Studies have shown that coating materials prepared by combining chitosan nanoparticles with methyl cellulose can significantly inhibit moisture loss and oil absorption during food processing, with respective reductions of 7.72% and 2.94% [11]. Moreover, a coating comprising linseed gum and carboxymethyl cellulose at a 1:1 ratio effectively suppressed acrylamide formation (relative reduction 36.61%) and lowered the oil content of fried potatoes by 37.98–39.46% compared with the control [12]. Notably, while hydrocolloids are widely used to regulate oil absorption in fried foods, most studies focus on direct coating with colloidal solutions. By contrast, studies that systematically investigate the effects of incorporating hydrocolloids into batter coatings on the oil penetration mechanism in fried products remain limited. Additionally, the impact of frying temperature on lipid oxidation in hydrocolloid-battered fish cubes and how these conditions regulate flavor compound formation pathways lack systematic theoretical understanding. This study aims to address these knowledge gaps, providing a comprehensive scientific basis for quality and flavor regulation in fried foods.

In this study, four typical hydrophilic colloids were incorporated into the batter system: guar gum, linseed gum, acacia senegal gum, and gellan gum. Through systematic measurements of batter rheological properties and viscosity parameters, the oil penetration mechanism and distribution patterns within the batter were deeply investigated. Furthermore, the effects of different frying temperatures on lipid oxidation degree and free fatty acid composition were explored to reveal the regulatory mechanism of hydrocolloids on flavor formation in battered fried fish cubes from the perspective of lipid oxidation. The research results of this study provide a systematic theoretical basis for the process optimization and flavor regulation of fried pre-prepared foods.

## 2. Results and Discussion

### 2.1. Effects of Different Hydrocolloids on the Rheological Properties of Batter

**Figure 1:**
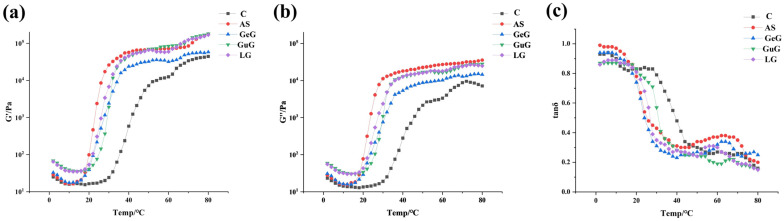
Rheological properties of batter coatings: loss modulus (**a**), storage modulus (**b**), and loss tangent (**c**). The abbreviations in this figure are defined as follows: C (control), guar gum (GuG), linseed gum (LG), acacia senegal gum (AS), and gellan gum (GeG).

This study first evaluated the effects of different hydrocolloids on the rheological properties of batter using rheological measurements. Specifically, the storage modulus was measured to characterize the material’s ability to store elastic deformation energy, reflecting the system’s elastic behavior. The loss modulus was determined to quantify energy dissipation under cyclic loading, revealing the system’s viscous characteristics. The ratio of loss modulus to storage modulus further illuminated the viscoelastic balance of the batter system. The experimental results revealed distinct regulatory effects of different hydrocolloids on the dynamic rheology of the batter, as illustrated in Figure 1.

The temperature-dependent evolution of G″ and G′ exhibited three distinct phases. In the initial stage, both G″ and G′ of the batter exhibited a gradual decline, indicating that the batter retained a certain degree of fluidity under relatively low heating temperatures. This reduction in viscoelastic moduli can be attributed to slight moisture loss and increased intermolecular spacing, which collectively diminished the system’s flow resistance. Subsequently, both G″ and G′ exhibited a trend of sharp increase followed by a gradual rise, and the batter with hydrocolloids (AS, GeG, GuG, LG) consistently maintained higher G″ and G′ values than the control (C) at the same temperature. Among the hydrocolloids, LG and AS groups exhibited relatively higher G″ values, suggesting they had a more pronounced effect on enhancing batter viscosity compared with GeG and GuG. This behavior confirmed that hydrocolloid incorporation enhanced the batter’s structural stability by facilitating the development of a more cohesive three-dimensional network [13,14]. Papagianni et al. [15] had similar research results; that is, flaxseed gum-enriched batters exhibited higher G″ and G′ values than the hydrocolloid-free control group. In the third stage, as the batter was completely solidified, both G′ and G″ tended to stabilize. Throughout the temperature sweep, all batters exhibited tanδ < 1, indicating that the batters exhibited predominantly elastic viscoelastic behavior. As the temperature increased, tanδ gradually decreased, and a sharp drop occurred when the temperature reached 20 °C. This characteristic point can be used as an indicator of gel point formation. Compared to the control, batters with added hydrocolloids showed reduced tanδ at 20–40 °C, suggesting that hydrocolloids promote gel protective layer formation by increasing viscosity, thereby inhibiting water loss and effectively blocking oil penetration [16].

### 2.2. Effects of Different Hydrocolloids on Batter Viscosity, Batter Pickup, Frying Yield, and Hardness

Batter pickup directly quantifies the adhesion capacity and deposition efficiency of batter, making it a pivotal parameter for evaluating the quality of battered and fried products. An adequate and uniformly distributed batter pickup not only ensures the formation of an intact, crispy crust during frying but also regulates the final oil absorption, textural properties, and overall sensory acceptability of the product. As indicated in Table 1, batter viscosity demonstrated a positive correlation with batter pickup, where higher viscosity was associated with greater adhesion of the batter onto the fish surface. Compared to the control, all four hydrocolloid batters showed significantly enhanced viscosity and pickup. Among them, the batter treated with LG exhibited the highest viscosity, followed by GuG, while AS exhibited relatively lower viscosity. Specifically, compared to the control group, the LG-supplemented group significantly increased batter viscosity by 74.9%, which in turn improved batter pickup by 26.1%. This difference may arise from the gel’s structural properties formed when hydrocolloids dissolve in water. The hydrocolloid gel network enhances batter adhesion, thereby improving batter pickup. Meanwhile, such a network structure significantly enhances the viscosity of the batter by promoting intermolecular interactions and reducing fluid mobility [17]. Notably, the core of viscosity differences observed among different hydrocolloids lies in the variations in their molecular composition, molecular weight, and chain structure [18]. Specifically, LG is composed of neutral and acidic polysaccharides, exhibits a broad molecular-weight distribution, and possesses moderately branched chains with high backbone flexibility; these features facilitate extensive intermolecular entanglement and thus confer high viscosity [19]. Consequently, this network promotes greater batter adhesion to fish surfaces, forming a thicker outer shell. This thicker coating helps regulate the frying process, ultimately contributing to increased frying yield. Frying yield comprehensively reflects the net result of moisture evaporation, oil penetration, and batter retention during frying. Overall, batter viscosity exhibits a positive correlation with frying yield, which is well supported by the results of the LG group. Specifically, the highest batter viscosity was displayed by the LG group, which also achieved the highest frying yield, an increase of 8.1% compared to the control group. Wang et al. [11] also reported that the frying yield of pork meatballs treated with carboxymethyl cellulose was significantly improved. Pong et al. [20] also confirmed that as the concentration of hydroxymethyl cellulose increased, the batter coating on the surface of chicken wings became thicker. In conclusion, the addition of hydrocolloids not only improves batter viscosity but also significantly enhances the batter pickup and frying yield in fried fish cubes.

Hardness is an important indicator for evaluating the quality of batter-coated fried foods, where a rigid and porous outer crust is pivotal in determining the crispy texture of the fried product. Table 1 demonstrates significant variations in hardness among treatment groups (*p* < 0.05), with the LG group exhibiting the highest hardness value, followed sequentially by the GuG, C, AS, and GeG groups. The colloidal structure of linseed gum and its conlinin protein content synergistically enhance the formation of a firm texture, likely due to increased disulfide bond cross-linking [21]. In the oil-rich frying medium, as the temperature increases, heat is transferred from the oil phase to the surface of batter-coated fish cubes and gradually conducted to the interior. During this process, the internal moisture of the fish cubes evaporates and is lost due to heating, while oil is continuously absorbed under the action of the internal–external pressure difference and capillary action [2]. This promotes the formation of a hard, porous, and crispy outer shell on the surface. Both oil and moisture migration influence food hardness. Batter coatings containing linseed gum or guar gum effectively inhibit the bidirectional migration of water and oil during frying, thereby enhancing the hardness of the outer shell [22]. This conclusion is consistent with the findings of Yu et al. [23], who reported that guar gum-coated potato chips exhibited higher hardness values. Conversely, fish cubes treated with acacia senegal gum and gellan gum showed lower hardness, presumably due to the migration of moisture from the fish meat to the batter layer during frying, leading to softening of the surface structure [24].

### 2.3. Effects of Different Hydrocolloids on Oil Absorption and Moisture Content

**Figure 2:**
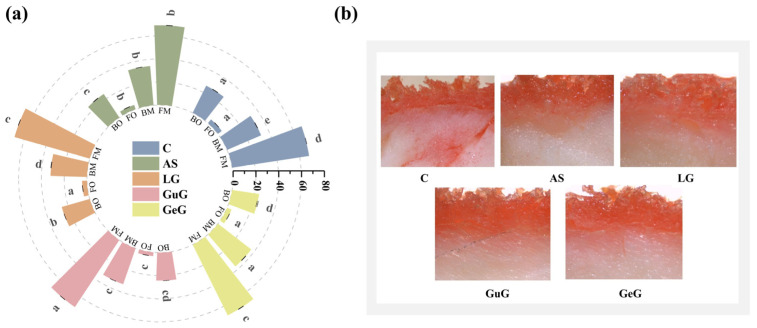
Changes in oil and moisture contents of fried fish cubes coated with batters containing different hydrocolloids (**a**). Oil penetration profiles in fried fish cubes (**b**). Abbreviations: Control (C), guar gum (GuG), linseed gum (LG), acacia senegal gum (AS), gellan gum (GeG), fish moisture (FM), batter moisture (BM), fish oil (FO), batter oil (BO).

The changes in oil and moisture content of battered fried fish cubes with different hydrocolloids are shown in Figure 2a. Compared to the control group, all hydrocolloid-based outer batter coatings exhibited significantly higher moisture content (*p* < 0.05). The GeG group demonstrated a 13.68% increase in moisture retention rate relative to the C group, with the AS group following closely. Conversely, hydrocolloid-treated batters had significantly lower oil content than the C group (*p* < 0.05). During frying, water and oil exhibit counterflow behavior: internal moisture evaporates and escapes due to elevated temperatures, while oil infiltrates the system via the combined effects of internal–external pressure gradients and capillary action [25]. Previous research has demonstrated that hydrocolloids improve batter system stability by facilitating the formation of an interactive filling film layer between starch and hydrocolloids within the protein network structure [26]. This mechanism effectively retards moisture evaporation and restricts oil penetration. In terms of oil content in fish meat, the AS and GuG groups exhibited significantly lower levels than the C group (*p* < 0.05), whereas no significant differences were observed between the LG and GeG groups and the C group (*p* > 0.05).

Additionally, batter viscosity exerts a bidirectional regulatory effect on the oil absorption process: excessively high viscosity tends to cause uneven distribution of batter particles, forming gaps that increase oil penetration pathways; conversely, excessively low viscosity leads to rapid moisture release during frying, promoting oil absorption through the water–oil displacement effect [27]. Thus, hydrocolloids with moderate viscosity optimize oil absorption in batters. Experimental results show that the viscosity of the GuG group is lower than that of the LG group, and its oil absorption is reduced by 9% compared with the C group. In contrast, the LG group exhibited relatively poor oil inhibition, likely due to high viscosity-induced particle aggregation, which created a rough surface and increased oil penetration pathways. Garmakhany et al. [28] reported that guar gum coating reduced the oil absorption rate of potato chips by 54.7% and increased the moisture content by 26.3%. Collectively, these findings indicate that hydrocolloids form an effective oil-control barrier by stabilizing and reinforcing the three-dimensional network structure of the outer batter coating.

### 2.4. Effects of Different Hydrocolloids on Oil Penetration in Fried Fish Cubes

To visually characterize oil penetration in battered fried fish cubes, the Sudan Red staining method was employed to observe oil distribution patterns in the samples. As shown in Figure 2b, there were differences in the degree of oil penetration between the control group and the hydrocolloid-treated groups. In the control group, Sudan Red staining revealed oil penetration into the fish meat, likely attributable to the insufficiently dense gel network formed by flour and starch alone, resulting in ineffective oil barrier properties. Furthermore, as shown in Table 1, the coating viscosity of the hydrocolloid-free group is relatively low. Lower viscosity weakens batter cohesion, which impairs its ability to form a thick, continuous, robust coating on fish cubes. As the frying process proceeds, capillary action can induce the formation of some small pores. More importantly, moisture within the food system evaporates rapidly under the high-temperature frying conditions. This water loss causes the outer thin batter layer to shrink, and the numerous large pores and cracks generated in this process collectively form the core channel structure that facilitates oil penetration into the food interior [29]. In contrast, the AS group showed that oil was primarily distributed in the outer shell layer and its junction with the fish cubes, with negligible oil penetration into the fish meat interior. The cross-linking interactions of hydrophilic groups in hydrocolloids with batter components form a dense network that inhibits moisture loss and oil penetration during frying [15]. In the LG, GeG, and GuG groups, limited oil migration into fish meat was observed, attributed to the insufficient stability of the gel networks formed by these hydrocolloids in the batter. Cui et al. [30] reported that xanthan gum-coated batters most effectively inhibited oil penetration, with Sudan Red staining confirming oil confinement to the outer shell layer. Collectively, these results show that selecting appropriate hydrocolloids can form an effective physical barrier, significantly reducing oil penetration. Notably, oil penetration depends not only on the batter microstructure but also on the physicochemical properties of the frying oil. Cui et al. [31] compared four common frying oils (rapeseed oil, soybean oil, rice bran oil, and palm oil) and found that the fish cubes fried in palm oil exhibited the highest oil content, reaching 14.2%. Palm oil’s high unsaturated fatty acid content fosters polar polymers, raising viscosity and lowering interfacial tension to enhance wettability and accelerate oil penetration into fried foods.

### 2.5. Effects of Different Heat Treatments on Lipid Oxidation of Fried Fish Cubes

**Figure 3:**
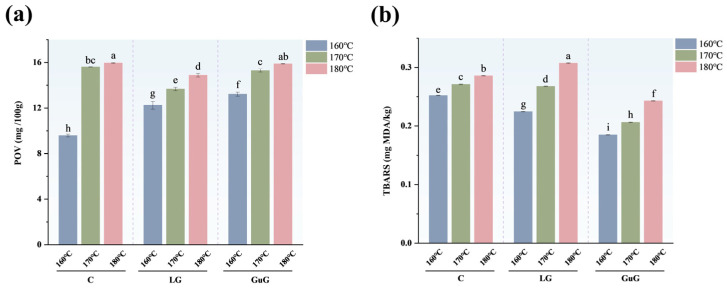
Oxidative indicators of battered fried fish cubes under different frying treatments. Peroxide value (POV) (**a**). Thiobarbituric acid reactive substances (TBARS) (**b**). Different superscript letters in the same row imply significant differences (*p* < 0.05). (*n* = 3, all measurements in triplicate). Abbreviations: Control (C), guar gum (GuG), linseed gum (LG).

Based on a comprehensive analysis of the above indicators, the LG and GuG groups with superior overall performance were selected for lipid oxidation analysis. TBARS and POV are key indicators for evaluating the degree of lipid oxidation in fried foods. Figure 3 shows the trends of POV and TBARS values in fish cubes at different frying temperatures. The results showed that the POV of fish cubes in the three treatment groups increased with the increase in frying temperature. As reported in relevant studies, oxidation products can form at a temperature of 150 °C, with a rise in frying temperature leading to an increase in hydrogen peroxide levels [32]. Although frying temperature increases peroxide levels, the overall POV remains low due to the low-fat content of fish meat, with oil primarily from batter-adsorbed frying oil. At 160 °C, the hydrocolloid group exhibited marginally higher POV than the control group. Notably, the control group at 160 °C exhibited a lower POV. The underlying mechanism for this phenomenon can be attributed to the following aspects. At 160 °C, all groups were in the initial stage of lipid oxidation induction, a stage characterized by a slow formation rate of hydroperoxides, and during this period, the POV was primarily determined by the decomposition rate of hydroperoxides [33]. The control group exhibited a relatively low viscosity, which not only facilitates the diffusion of hydroperoxides and their subsequent catalytic decomposition by metal ions but also enables more uniform heat transfer in its system, further accelerating the thermal decomposition of hydroperoxides [34]. However, at 170 °C and 180 °C, a significant reduction in POV was observed in LG hydrocolloid-treated samples compared to controls. Specifically, the LG-180 °C group had a POV of 14.88 mg/100 g, which was slightly lower than that of the C-180 °C group (15.95 mg/100 g). At relatively high frying temperatures, the structured layer formed by hydrocolloids can effectively inhibit oxygen diffusion into the sample interior, thereby reducing the formation rate of hydroperoxides within the system. Additionally, hydroperoxides tend to decompose into secondary oxidation products (e.g., aldehydes and ketones) under high temperatures, which consequently leads to a lower POV [9].

The TBARS content is an indicator reflecting the degree of secondary lipid oxidation. As shown in Figure 3b, the TBARS content of the three groups of batter-coated fish cubes increased with the rise in frying temperature. The LG group exhibited a modest reduction in TBARS relative to the control at 160 °C and 170 °C but slightly surpassed it at 180 °C (peak 0.31 mg MDA/kg). The GuG group showed a smaller TBARS decrease, consistent with its marginally lower oil content in both fish muscle and batter. Jouki et al.’s research confirmed that hydrocolloids like papaya seed gum reduce TBARS in fried chicken, likely by forming a barrier to inhibit oil adsorption and delay oxidation [9]. These findings indicate that hydrocolloids’ regulation of secondary lipid oxidation depends on frying temperature and hydrocolloid type.

### 2.6. Effects of Different Heat Treatments on Sensory Analysis of Fried Fish Cubes

**Figure 4:**
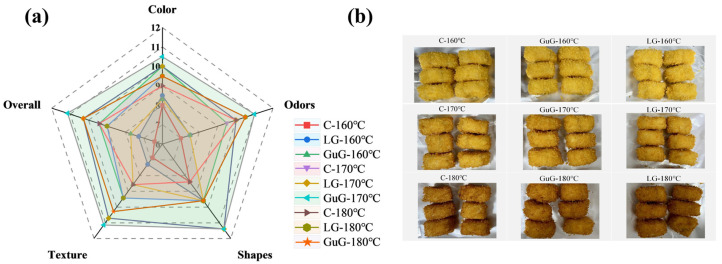
Sensory indicators of battered fried fish cubes under different frying treatments. Sensory evaluation radar chart (**a**). Optical photographs of fried fish samples (**b**). Abbreviations: Control (C), guar gum (GuG), linseed gum (LG).

In this study, a sensory evaluation panel assessed batter-coated fried fish cubes under different treatments using a 1–12 point scale for color, texture, appearance, odor, and overall acceptability. As shown in Figure 4a, among the three frying temperatures, the fish cubes treated at 170 °C had the largest polygon area in the sensory profile, with the highest scores in all indices, indicating optimal overall sensory quality at this temperature. Among them, the GuG-170 °C group nearly achieved the full score in color evaluation, showing a prominent advantage. Color scores showed no obvious difference between the C group and the LG-170 °C group. In addition, based on Figure 4b, the fish cubes treated at 180 °C showed burnt outer shells due to over-frying, leading to lower color scores. The golden color of the fried fish cubes’ outer shell results from the combined effects of moisture content, oil–material interactions, Maillard reactions, and non-enzymatic browning. Optimizing these factors can enhance the color performance of fish cubes. Regarding texture, fish cubes fried at 170 °C and 180 °C exhibited crispness, with the GuG-170 °C group showing the most outstanding texture performance, followed by the LG-170 °C group. In contrast, those fried at 160 °C lacked crispness due to the lower temperature, resulting in a softer texture. Particularly, the C-160 °C group had the lowest score. Considering comprehensive sensory indices, including color and texture, the GuG-170 °C group achieved the highest overall acceptability score. This confirms that under this temperature condition, batter-coated fish cubes with added hydrocolloids can effectively enhance sensory quality.

### 2.7. Effects of Different Heat Treatments on FFA in Fried Fish Cubes

**Figure 5:**
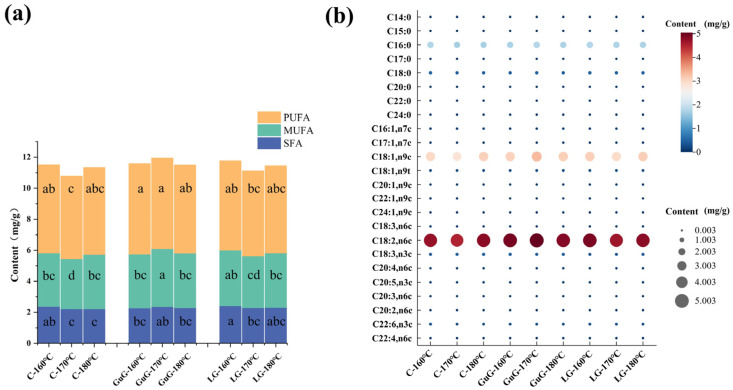
Compositional profiles of fatty acids in battered fried fish cubes under different frying treatments. Stacked bar chart of the composition of fatty acid classes (**a**). Bubble plot of individual fatty acid concentrations (**b**). Abbreviations: Control (C), guar gum (GuG), linseed gum (LG), polyunsaturated fatty acid (PUFA), monounsaturated fatty acid (MUFA), saturated fatty acid (SFA).

FFA, as precursors of numerous flavors, are crucial for constructing food flavor profiles. Due to the unsaturated bonds in their molecular structure, unsaturated fatty acids are more susceptible to oxidative degradation than saturated fatty acids during processing and storage. Through gradual oxidation, unsaturated fatty acids decompose into small-molecule volatile compounds (aldehydes, ketones, alcohols), which impart unique aromas to foods. Thus, unsaturated fatty acids play a pivotal role in the flavor formation and quality improvement of batter-coated fried fish cubes, serving as an essential material basis for regulating product flavor characteristics [32]. As shown in Figure 5b, a total of 24 free fatty acids were identified in battered fried fish cubes, including 8 saturated fatty acids (SFAs), 7 monounsaturated fatty acids (MUFAs), and 9 polyunsaturated fatty acids (PUFAs). In fried fish cubes, PUFAs were the most abundant, constituting over 49% of the total FFA. MUFAs were the second most prevalent, accounting for more than 30%. In contrast, SFAs were relatively less abundant, comprising approximately 19% of the total FFA. Among them, methyl palmitate (C16:0) and methyl stearate (C18:0) were the main saturated fatty acid components, whereas methyl linoleate (C18:2, n6c) and methyl oleate (C18:1, n9c) were the primary polyunsaturated fatty acid constituents.

As shown in Figure 5a, unsaturated fatty acids (UFAs) were the predominant fatty acid components in batter-coated fried fish cubes. The SFAs content in the C and LG groups generally decreased with the increase in frying temperature, and there was no obvious difference between the 170 °C and 180 °C treatment groups. However, the SFA content in the GuG group showed a decreasing trend at 180 °C. MUFAs and PUFAs contents in the C and LG groups declined at 170 °C, whereas those in the GuG group only decreased at 180 °C. This trend is related to the temperature response characteristics of fatty acid components and the regulatory effect of hydrocolloids. Islam et al. [35] similarly showed that β-sitosterol-added fried fish surimi had higher UFA content than controls. Overall, the fatty acid content of fish cubes varied considerably across different frying treatments. Treatments at C-170 °C, CuG-180 °C, and LG-170 °C reduced UFA content to different extents, implying potential impacts on the battered fried fish cubes’ flavor.

### 2.8. Analysis of Volatile Components in Battered Fried Fish Cubes

**Figure 6:**
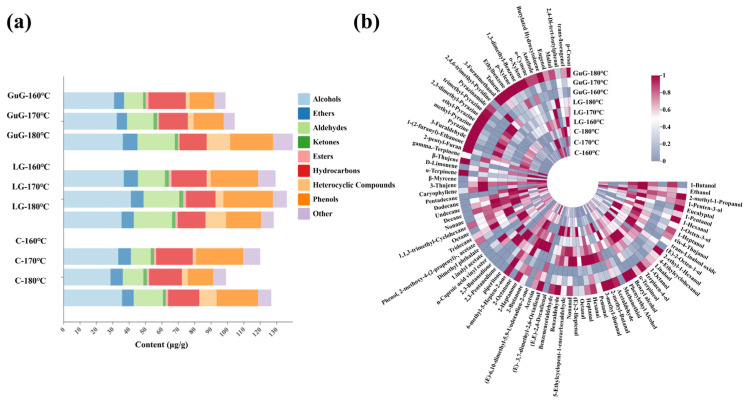
Compositional profiles of volatile flavor compounds in battered fried fish cubes under different frying treatments. Stacked bar chart of the composition of volatile flavor compound classes (**a**). Heatmap of volatile compounds (**b**). Abbreviations: Control (C), guar gum (GuG), linseed gum (LG).

GC-MS analysis identified 87 volatile compounds in batter-coated fried fish cubes, including 18 alcohols, 2 ethers, 14 aldehydes, 9 ketones, 4 esters, 21 hydrocarbons, 11 heterocyclic compounds, 4 phenols, and 4 other compounds. The flavor compound category bar chart and group content heat map are presented in Figure 6.

As shown in Figure 6a, the LG group exhibited the highest alcohol content. In the GuG group, total alcohol content increased with frying temperature, peaking at 180 °C. Combined with Figure 6b, major alcohols in fried fish cubes included ethanol, 1-hexanol, 1-octen-3-ol, linalool, α-terpineol, and phenethyl alcohol. Although alcohol compounds generally have high sensory thresholds, they can still contribute to food flavor at elevated concentrations. Meanwhile, alcohol also interacts with other flavor compounds, modulating overall flavor profiles. For example, research by Cameleyre et al. [36] has shown that higher alcohols in alcoholic model systems enhanced butyric acid perception while suppressing fruity aromas. 1-octen-3-ol, a typical unsaturated alcohol in fish meat, exhibits mushroom, hay, and rose aromas, primarily derived from the catabolism of linoleic acid and arachidonic acid. In this study, 1-octen-3-ol content increased with frying temperature, peaking in the LG-170 °C group. Additionally, saturated alcohols such as 1-pentanol, 1-heptanol, 4-thujanol, and 4-ethylcyclohexanol also rose with temperature, likely due to high-temperature promotion of unsaturated fatty acid oxidative decomposition, generating more alcohol products.

In all fried fish cube groups, aldehyde concentration increased with frying temperature. Linear aldehydes such as pentanal, heptanal, octanal, nonanal, and acetaldehyde primarily originated from fatty acid oxidative degradation, contributing grassy and fruity aromas. These linear aldehydes, characterized by high concentrations and low sensory thresholds, were pivotal in shaping the overall flavor of fried fish cubes. Aldehydes also form via protein oxidation pathways—for instance, benzaldehyde, which imparts nutty aromas to meat products, arises from Strecker degradation [37]. Among unsaturated aldehydes, (*E*)-2-heptenal has a green vegetable aroma and serves as a secondary oxidation product of linoleic acid. The (*E*)-2-heptenal content in the control group exceeded that in the GuG group, likely due to greater oil adsorption in control samples during frying, which promoted more oxidative degradation of linoleic acid. (*E*, *E*)-2,4-Decadienal, exhibiting a strong chicken-fat-like aroma, is formed via high-temperature degradation and oxidation of lipids [38]. Overall, hydrocolloid-battered fish cubes treated at 170 °C and 180 °C showed more prominent aldehyde-derived flavors, particularly the LG-180 °C group. In conclusion, aldehydes generated by thermal oxidation of lipids and proteins make substantial contributions to flavor formation in fried fish cubes.

Heterocyclic compounds are primarily generated via the Maillard reaction and carbohydrate degradation, typically exhibiting characteristic baking, roasting, nutty, and caramel aromas. Due to their low aroma thresholds, they make important contributions to the overall flavor of fried fish cubes. Heterocyclic compounds detected in fried fish cubes mainly include pyrazines, furans, and pyridines. Among them, pyrazine compounds form through reactions between carbonyl fragments from sugar cleavage and amine groups from amino acid degradation, while furan compounds primarily originate from high-temperature carbohydrate degradation and lipid oxidation processes [2]. As shown in the Supplementary Table S1 [39,40,41,42], the content and types of heterocyclic compounds increased with frying temperature, peaking at 180 °C. Notably, pyrazine, ethyl-pyrazine, 2,3-dimethyl-pyrazine, and pyrazinamide were detected exclusively in the 170 °C and 180 °C treatment groups. Heterocyclic compound content was higher in battered fried fish cubes than in the control group, with the GuG-180 °C group exhibiting the highest content. This may be attributed to the high viscosity of hydrocolloids, which enhanced batter adhesion to fish cubes and increased Maillard reaction substrate concentration. Previous studies have shown that excessively high oil temperatures can induce Maillard reactions that adversely affect product quality, such as promoting the formation of advanced glycation end products [38]. Sensory evaluation results also indicated that battered fried fish cubes at 180 °C had obvious burnt flavors. Thus, considering both flavor quality and safety, 180 °C is unsuitable as the frying temperature for battered fried fish cubes.

Ketones primarily derive from fatty acid oxidation and amino acid degradation. Due to their high sensory thresholds, they make minimal direct contributions to overall flavor. With increasing frying temperature, ketone content exhibited a trend of first increasing and then decreasing, suggesting that ketones may serve as flavor precursors in Maillard reactions, converting into more volatile flavor substances [43]. Acetoin, a common methyl ketone in meat products, imparts a buttery aroma, with formation pathways including amino acid decomposition, fatty acid autoxidation, or β-oxidation [44]. Esters, which impart fruity flavors to meat products, are produced by esterification reactions between free fatty acids and alcohols generated from lipid oxidation. In this study, a few esters were detected in fried fish cubes, mainly including vinyl caproate, dimethyl phthalate, and linalyl acetate. Esters typically have low sensory thresholds and play an important role in food flavor. Hydrocarbons mainly originate from lipid degradation. The hydrocarbons detected in this study included limonene, α-terpinene, β-myrcene, caryophyllene, dodecane, and undecane. With increasing temperature, the content of hydrocarbons decreased, and the content in the hydrocolloid group was generally lower than that in the control group. Most hydrocarbons exhibit sweet and aromatic flavors, but due to their high thresholds, they contribute little to the flavor of fish cubes [45].

In summary, frying at 170 °C and 180 °C promotes the release of flavor compounds. However, frying at 180 °C induces excessive Maillard reactions and lipid oxidation, leading to undesirable burnt flavors. Therefore, 170 °C is the optimal processing temperature for battered fried fish cubes. Moreover, hydrocolloid-battered fish cubes exhibited superior flavor compared to the control group, with the LG group showing the most pronounced effect. This can be attributed to hydrocolloids strengthening the batter’s cross-linked structure and forming a protective film around flavor compounds, thereby mitigating their degradation during frying [46].

### 2.9. Correlation Analysis Between Characteristic Flavor Compounds and Lipid Oxidation

**Figure 7:**
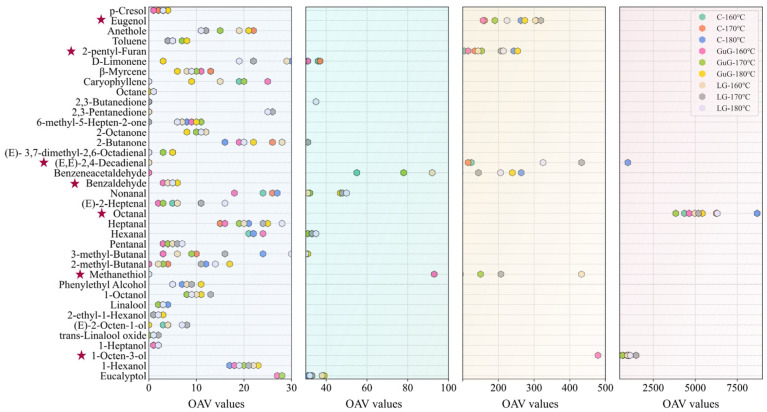
Odor activity values of key flavor compounds with OAV ≥ 1 (compounds with OAV ≥ 100 are marked with a red star). Abbreviations: Control (C), guar gum (GuG), linseed gum (LG), Odor Activity Value (OAV).

To further explore the key aroma-contributing compounds of fried fish cubes, this study conducted OAV analysis for aroma compounds. A compound with an OAV ≥ 1 is considered to make a substantial contribution to the overall aroma of battered fried fish cubes. As shown in Figure 7, 37 flavor components exhibited OAV ≥ 1. Among these, seven key volatile compounds with OAV ≥ 100 were identified as major contributors to the overall flavor profile. These include eugenol (clove-like aroma), 2-pentylfuran (bean-like), (*E*, *E*)-2,4-decadienal (oily/fatty), octanal (citrusy), methanethiol (sulfurous/rotten egg-like), and 1-octen-3-ol (mushroom-like). As shown in Figure 4b, aldehydes and alcohols constituted the major components among compounds with OAV ≥ 1. Research has established that short-chain aldehydes (hexanal, pentanal) impart characteristic grassy notes, while their longer-chain counterparts develop progressively stronger fatty aromas. Notably, branched-chain aldehydes (2-methylbutanal and 3-methylbutanal), predominantly derived from amino acid degradation, contribute distinct nutty flavor profiles [47]. Among aldehydes, octanal had the highest OAV, mainly derived from the oxidative decomposition of oleic acid. The C-180 °C group showed the highest octanal content, consistent with the abundant reaction substrates. Among alcohols with OAV ≥ 1, 1-octen-3-ol exhibited a high OAV, featuring a typical mushroom aroma. This compound is abundantly detected in heat-treated vegetable oils and has been confirmed as a characteristic decomposition product of linoleic acid [48].

**Figure 8:**
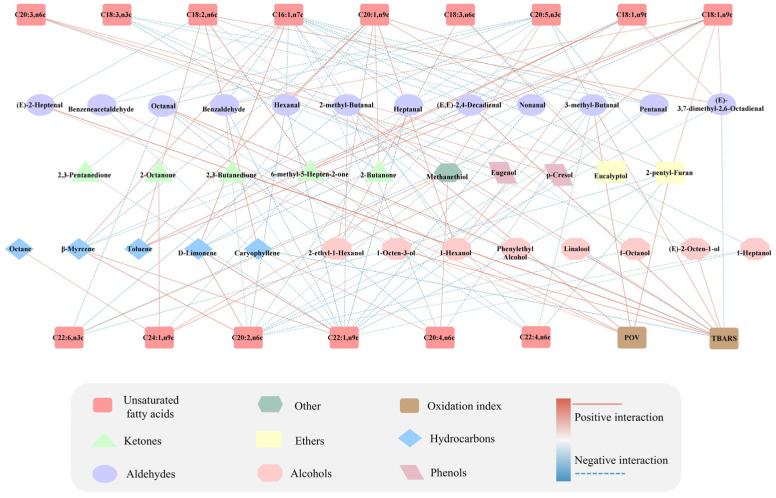
Correlation analysis between key flavor compounds, unsaturated fatty acids, and oxidation indicators.

Using a correlation coefficient |R| ≥ 0.5 as the screening criterion, Spearman correlation analysis was performed between characteristic flavor compounds with OAV ≥ 1 and lipid oxidation factors. As shown in Figure 8, methanethiol with high OAV exhibited strong positive correlations with multiple PUFAs, including C24:1, n9c; C20:4, n6c; C20:5, n3c; and C22:6, n3c. This result indicates a close association between methanethiol formation and the oxidation process of fatty acids. In fried food systems, methanethiol is primarily generated via the Strecker degradation of methionine, and free radicals from PUFA oxidation may promote methionine breakdown, thus accelerating methanethiol formation [49]. Additionally, pentanal showed positive correlation with specific fatty acids, such as C20:1, n9c, but negative correlations with C22:1, n9c; C18:3, n3c; and C20:2, n6c. This may be attributed to structural differences in fatty acids (such as chain length and double bond positions) and distinct oxidation pathways.

Most compounds exhibited strong positive correlations with POV or TBARS. For instance, 1-octen-3-ol, linalool, (*E*, *E*)-2,4-decadienal, and 2,3-butanedione showed positive correlations with POV. Octanal, (*E*)-2-heptenal, and 2-methylbutanal exhibited positive correlations with both POV and TBARS values. These findings demonstrate that lipid oxidation plays a pivotal role in flavor development in fried fish cubes. Thermal-induced radical formation initiates oxidative chain reactions that generate key volatile flavor compounds, including alcohols, aldehydes, and ketones [48].

### 2.10. Analysis of the Potential Formation Pathways of Characteristic Flavor Compounds

**Figure 9:**
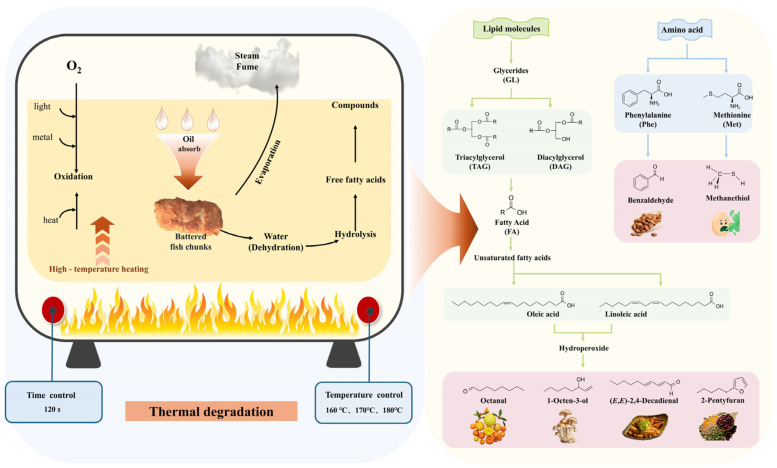
Analysis of the potential formation pathways of characteristic flavor compounds.

A preliminary analysis of the formation pathways of characteristic aroma compounds (OAV ≥ 100) was conducted, with the potential metabolic pathways illustrated in Figure 9. Eugenol was detected in fried samples across three variants of batter-coated fish cubes at different temperatures. As eugenol is a signature component of clove, processing analysis suggests its origin may be associated with the clove content in the shisanxiang spice blend used for fish deodorization [50]. The formation of the remaining five key aroma compounds primarily involves lipid oxidation and amino acid degradation. Lipid oxidation during the frying of batter-coated fish cubes mainly comprises thermal oxidation and auto-oxidation, both following similar reaction processes with three stages: initiation, propagation, and termination [51]. The lipid oxidation rate increases significantly with rising temperature, accompanied by a corresponding increase in volatile compound release. When the temperature reaches the smoke point of the oil, volatile decomposition products are released as visible fumes, signaling thermal degradation of triglycerides into glycerol and free fatty acids.

Glycerides (GL) are typically classified into triglycerides (TAG) and diglycerides (DAG) based on the number of fatty acids they contain. Under the influence of moisture and high temperatures, the ester bonds in GL undergo hydrolysis, yielding free glycerol and fatty acids (FA) [6]. Although most glycerol is volatile at 150 °C, the remaining glycerol can further promote the generation of free FAs through additional hydrolysis [51]. Among these, UFAs such as oleic acid and linoleic acid can further generate hydroperoxides, which ultimately decompose into volatile flavor compounds such as aldehydes and alcohols. Octanal, exhibiting a citrus-like fruity aroma, is an oxidation product of MUFAs such as oleic acid. 1-octen-3-ol is generated from the 10-hydroperoxide of linoleic acid and other unsaturated fatty acids [52]. (*E*, *E*)-2,4-Decadienal and 2-pentylfuran originate from the 9-hydroperoxide of linoleic acid and other unsaturated fatty acids [53]. Additionally, amino acids released through protein degradation during frying act as key flavor precursors. Methionine undergoes Strecker degradation to form methional, which subsequently decomposes into methanethiol. Phenylalanine can be converted to benzaldehyde via direct synthesis through Maillard reaction intermediates, including Strecker degradation products [49].

## 3. Conclusions

Hydrocolloids interact with flour to form a stable network structure, increasing system viscosity and thus improving the batter pickup and frying yield of fried fish cubes. Rheological tests demonstrate that hydrocolloids significantly increase storage and loss moduli, improving batter stability. Oil content analysis and Sudan Red staining reveal that guar gum significantly reduces oil absorption compared to the control group, with oil predominantly localized in the outer shell layer. As frying temperature increases, lipid oxidation deepens, but hydrocolloids significantly slow this process. Sensory evaluation confirms that GuG-170 °C fish cubes have the crispest texture and the highest overall score. Fatty acid analysis indicates that hydrocolloids and frying temperature jointly regulate free fatty acid content. GC-MS identifies 78 volatile flavor compounds, with lipid oxidation as the main pathway for key aroma compounds. Hydrocolloid-battered fish cubes effectively release flavor compounds at 170 °C and 180 °C. However, excessive lipid oxidation and Maillard reactions at higher temperatures may produce undesirable flavors, making 170 °C a more suitable processing temperature. Overall, the GuG-170 °C treatment reduces oil absorption, improves sensory quality, and promotes flavor release, making it an optimal choice for batter-fried fish cubes. While this study centered on screening different types of hydrocolloids, it did not explore how varying concentrations of these hydrocolloids influence the fried fish cubes. Future research should first determine the optimal hydrocolloid dosage and then couple this optimized condition with green processing technologies (e.g., air frying) to further enhance the product’s health value.

## 4. Materials and Methods

### 4.1. Materials and Reagents

Sea bass (*Lateolabrax japonicas*) weighing 500–600 g and measuring 28–32 cm in total length were purchased alive from a perch aquaculture base in Zhuhai and transported to the laboratory. Arawana Golden Ratio vegetable oil (composed of 49% soybean oil, 21% rapeseed oil, 14% sunflower seed oil, 9% corn oil, 3% peanut oil, 3% rice bran oil, 0.6% sesame oil, and 0.4% flaxseed oil), salt, white pepper, Wang shouyi Shi San Xiang spice blend (containing Sichuan pepper, star anise, fennel, angelica, clove, nutmeg, cinnamon, amomum, cardamom, costus root, sand ginger, galangal, and dried ginger), ginger, cooking wine, Sichuan pepper, gluten flour, sweet potato flour, glutinous rice flour, panko, and eggs were procured from local supermarkets; sodium tripolyphosphate was obtained from Guangdong Jianda Biotechnology. Guar gum (GuG), acacia senegal gum (AS), linseed gum (LG), and gellan gum (GeG) were supplied by Guangzhou Xinruorong Biotechnology Co., Ltd. (Guangzhou, China). The above-mentioned materials were used for the preparation of raw materials for fish cubes coated with batter prior to frying.

Analytical-grade reagents included petroleum ether and chloroform (Guangzhou Chemical Reagent Factory, Guangzhou, China); trichloroacetic acid (Shanghai Aladdin, Shanghai, China); glacial acetic acid (Guangzhou Jinyuan Chemical, Guangzhou, China); potassium iodide, sodium thiosulfate, anhydrous sodium sulfate, and soluble starch (Shanghai Macklin, Shanghai, China); disodium EDTA (Sinopharm Group, Shanghai, China); and thiobarbituric acid (Shanghai Yuanye, Shanghai, China). N-hexane and 14% boron trifluoride-methanol solution were from Sigma-Aldrich, Saint Louis, MO, USA, and a 37-component fatty acid mixture was from Merck (Darmstadt, Germany). All reagents were of analytical grade. All reagents employed were of analytical grade, and they were used for the determination of experimental indicators, including lipid oxidation and fatty acid content.

### 4.2. Sample Preparation

The marinade for sea bass cubes was formulated as a percentage of fish mass: 7 wt% salt, 0.5 wt% sodium tripolyphosphate, 0.2 wt% white pepper, 0.1 wt% Shisanxiang, 2 wt% cooking wine, 2 wt% ginger, 0.5 wt% Sichuan pepper, and 100 wt% ice water (ice: water = 1:1). The batter recipe comprised 0.4 g of the respective hydrocolloid (guar gum, linseed gum, acacia senegal gum or gellan gum), 50 g of wheat flour, 50 g of glutinous rice flour, 25 g of sweet potato flour, 0.5 g of baking soda, 0.5 g of sodium pyrophosphate, 65 g of egg liquid (whole egg), and 65 g of water. The main objective of this experiment was to investigate the effects of different hydrocolloids on fried fish cubes. Accordingly, only the differences among various hydrocolloid types at a uniform addition concentration (0.4 g) were evaluated, and no additional concentration gradients were set to explore the dose–effect relationship. Low-gluten flour, glutinous rice flour, sweet potato flour, baking powder, and hydrocolloid were mixed, followed by adding water and egg liquid. The mixture was homogenized at 1000 r/min for 5 min using a T25 homogenizer (IKA, Staufen, Germany) to prepare a uniform batter.

The marinade and fish cubes were placed in a vacuum tumbler (Guangdong Adibao, Guangzhou, China) and tumbled at medium speed for 30 min under atmospheric pressure. After marination, the fish was blotted dry, cut into 12 g cubes (3 × 1.5 × 1 cm^3^), dipped in the prepared batter until evenly coated, and lifted with chopsticks to drain excess batter. The battered cubes were then coated with breadcrumbs. Frying was conducted in 3 L of Arawana Golden Ratio vegetable oil preheated to 170 °C in an XJ-7K115 fryer (Hubei Xiangjiang Electric Appliance Co., Ltd., Huanggang, China). Each batch (≤9 cubes) was fried for 2 min, then removed and air-cooled to 50 °C before being subjected to analysis. Fresh oil was used for each trial. Treatment groups were labeled as follows: acacia gum (AS), gellan gum (GeG), guar gum (GuG), linseed gum (LG), with the hydrocolloid-free control denoted as C.

### 4.3. Viscosity Measurement of Batter

The viscosity of batters containing different hydrocolloids was measured using an NDJ-8S viscometer (Lichen Bangxi Instrument Technology Co., Ltd., Shanghai, China) equipped with a No. 3 rotor at a rotational speed of 12 r/min.

### 4.4. Rheological Properties of Batter

The rheological properties of the batter were measured using an HR-1 rheometer (TA Instruments, New Castle, DE, USA), with the method referenced from Lin et al. [54] and modified accordingly. Temperature sweep measurements were conducted by depositing 2 mL of batter onto a 60 mm diameter, 1.016° cone-plate geometry. The cone was lowered with a 1 N contact force to ensure full sample contact, followed by heating from 0 °C to 80 °C at 2 °C/min. The dynamic viscoelastic parameters, including loss modulus (G″), storage modulus (G′), and loss tangent (tanδ = G″/G′), were systematically recorded during testing.

### 4.5. Determination of Batter Pickup and Frying Yield

The initial fish weight was recorded as *m* (g). The combined weight of the batter and container for holding the batter before coating was *m*_1_ (g), and after coating, the remaining batter and container weighed *m*_2_ (g); frying yield was calculated following the method of Park et al. [55]. The breadcrumb-coated fish cubes were weighed as *m*_3_ (g) before frying and *m*_4_ (g) after frying. The calculation formulas for batter pickup and frying yield are as follows:

(1) $$Batter\;pickup(\%)=\frac{m_{2}-m_{1}}{m}\times 100$$

(2) $$Frying\;yield\;(\%)=\frac{m_{4}}{m_{3}}\times 100$$

### 4.6. Determination of Oil and Moisture Content

The outer coating and fish meat of fried fish cubes were separated and ground for subsequent analysis. Oil content in the coating and moisture content in the fish meat were determined using a fat extractor (SOX406, Haineng Instruments, Jinan, China) and a moisture analyzer (MA35, Sartorius, Goettingen, Germany), respectively.

### 4.7. Determination of Hardness

The hardness of the battered fish cubes was determined using a CT3-4500 Texture Analyzer (Brookfield, New York, NY, USA) according to Zhang et al. [56]. The parameters were set as follows: TA44 probe, test speed of 1.0 mm/s, trigger force of 0.5 g, compression distance of 5.00 mm, and single-cycle measurement.

### 4.8. Sudan Red Dyeing of Fried Battered Fish Cubes

Following the method of Zhai et al. [57], 1.0 g of Sudan Red B was accurately weighed and added to 2 L of Arawana Golden Ratio vegetable oil; the mixture was then incubated in a water bath at 60 °C for 4 h to ensure complete dissolution of Sudan Red B. Coated fish cubes were fried in the dyed oil, cooled, and frozen at −18 °C for 5 h. The cubes were then sectioned into 3 mm slices, and their cross-sections were observed under an optical microscope at 4× magnification to assess dye penetration.

### 4.9. Determination of Malondialdehyde and Peroxide Value

The pretreatment of samples before frying followed Section 4.2. Battered fish from the control, guar gum, and linseed gum groups were deep-fried at 160, 170, or 180 °C for 2 min with precise temperature control. After frying, samples were immediately removed, cooled to room temperature (25 ± 2 °C), and homogenized for uniformity before analysis. Thiobarbituric Acid Reactive Substances (TBARS), an indicator of lipid oxidation based on malondialdehyde (MDA) content, and Peroxide Value (POV) in the fish cubes were determined according to Chinese National Standards: GB 5009.181-2016 [58] for MDA and GB 5009.227-2016 [59] for POV.

### 4.10. Sensory Evaluation

A sensory evaluation panel comprising 10 trained food science students (5 male, 5 female; 22–28 years old) was established. Panelists evaluated battered fish cubes under different frying treatments using a 12-point hedonic scale (1 = extremely dislike, 12 = extremely like). Specific scoring criteria are detailed in Table 2. The sensory properties are a comprehensive reflection of the chemical and physical changes that occur during frying. For instance, a uniform golden color indicates a moderate extent of the Maillard reaction, while the crispy coating and the juicy texture within the fish cubes reflect the synergistic effects of multiple physical indicators, including the hardness and water-holding capacity of the battered and fried fish cubes.

### 4.11. Determination of Free Fatty Acid

The frying conditions for the fish cubes are as specified in Section 4.9. Free fatty acid (FFA) content in fish cubes was determined according to Chinese National Standards: GB 5009.168-2016 [60].

### 4.12. Determination of Volatile Flavor Compounds

Sample preparation followed the protocol described in Section 4.11. Volatile flavor compounds in the fried fish samples were extracted using a headspace solid-phase microextraction (HS-SPME) device (57330-U, Supelco, Bellefonte, PA, USA). Subsequent qualitative and quantitative analyses were performed by gas chromatography–mass spectrometry (GC-MS) (Agilent 7890A-5975C, Agilent Technologies, Santa Clara, CA, USA). Volatile flavor compounds were analyzed using a method based on Nie et al. [61].

The heating procedure for the DB-5MS column (30 m × 0.25 mm × 0.25 μm) was as follows: 40 °C for 5 min, then ramped to 240 °C at 5 °C/min, and finally ramped to 250 °C at 10 °C/min for 6 min. The mass spectrometer was operated in the electron impact mode at an electron energy of 70 eV, with a mass range of 29–550 Da.

The Odor Activity Values (OAVs) of the aroma compounds were calculated to evaluate the key aroma compounds. The OAV is calculated based on the ratio of the relative content (C, mg/kg) of the aroma compounds to their threshold value (T, mg/kg). The calculation formula for OAV is as follows:

(3) $$OAV=\frac{C}{T}$$

### 4.13. Statistical Analysis

All experiments were performed with at least three independent replicates. Statistical analysis was conducted using one-way ANOVA in SPSS 26 (SPSS Inc., Chicago, IL, USA), with a statistical significance level of 0.05. Data visualization was performed using Origin (Origin Lab, Northampton, MA, USA), TBtools-II (https://github.com/CJ-Chen/TBtools (accessed on 10 July 2024)), and ChiPlot (https://www.chiplot.online/ (accessed on 15 June 2024)).

## Figures and Tables

**Table 1:** Effects of different hydrocolloids on batter viscosity, batter pickup, frying yield, and hardness.

Group	Batter Viscosity (mPa s)	Batter Pickup (%)	Frying Yield (%)	Hardness (g)
C	2710 ± 8.19 ^e^	16.70 ± 0.18 ^e^	123.85 ± 0.84 ^e^	127.33 ± 2.52 ^c^
AS	2946 ± 15.72 ^d^	17.57 ± 0.12 ^d^	126.83 ± 0.72 ^d^	112.67 ± 2.52 ^d^
LG	4740 ± 8.08 ^a^	21.05 ± 0.58 ^a^	133.91 ± 0.11 ^a^	160.00 ± 5.00 ^a^
GuG	4154 ± 7.94 ^b^	19.56 ± 0.41 ^b^	131.62 ± 0.30 ^b^	142.67 ± 5.13 ^b^
GeG	3546 ± 15.28 ^c^	18.47 ± 0.37 ^c^	129.35 ± 1.48 ^c^	104.00 ± 2.65 ^e^

Note. Different superscript letters in the same row imply significant differences (*p* < 0.05). Abbreviations: Control (C), guar gum (GuG), linseed gum (LG), acacia senegal gum (AS), and gellan gum (GeG).

**Table 2:** Sensory evaluation criteria for battered fried fish cubes.

Evaluation Attributes	1~4 Points	5~8 Points	9~12 Points
Color	Excessive browning or a pale white appearance on the coating	Relatively uniform color with slight browning on the coating	Uniform golden yellow color on the coating
Shapes	Cracks on the coating or obvious breadcrumb shedding	Slight breadcrumb shedding	Intact fish cubes
Odors	Obvious burnt odor or lack of aroma	Relatively rich aroma with slight burnt odor	Rich aroma with complex layers
Texture	Non-crispy coating, dry fish meat	Moderately crispy coating	Crispy coating, juicy fish meat
Overall	Poor overall experience, disliked	Average overall experience	Extremely pleasant overall experience

## Data Availability

The original contributions presented in this study are included in the article. Further inquiries can be directed to the corresponding author.

## Supplementary Materials

The following supporting information can be downloaded at: https://www.mdpi.com/article/10.3390/gels11100781/s1; Table S1: Flavor compounds of battered fried fish cubes under different frying treatments.
