# Supplementary material for: Regulation of Oil Penetration, Lipid Oxidation, and Flavor Characteristics in Batter-Coated Fried Fish Cubes: The Functional Implications of Hydrocolloids
Source: Gels. 2025 Sep 30;11(10):781. doi: 10.3390/gels11100781 (PMC12562527; doi:10.3390/gels11100781)
Supplement: Supplementary file 1 [file gels-11-00781-s001.zip › gels-3853015-supplementary.pdf]

Table S1. Flavor compounds of battered fried fish cubes under different frying treatments

No.	Category	Compounds	Threshold (mg/kg)	Content (ug/g)								
				C-160℃	C-170℃	C-180℃	LG-160℃	LG-170℃	LG-180℃	GuG-160℃	GuG-170℃	GuG-180℃
1	Alcohols	1-Butanol	0.5 [a]	0.07	0.00	0.00	0.08	0.09	0.07	0.00	0.08	0.00
2		Ethanol	2900 [a]	20.58	15.75	20.42	21.83	23.17	20.40	20.06	20.67	19.04
3		2-methyl-1-Propanol	8 [a]	0.22	0.19	0.23	0.00	0.16	0.20	0.18	0.16	0.23
4		1-Penten-3-ol	3 [a]	0.00	0.12	0.18	0.23	0.28	0.20	0.11	0.20	0.22
5		1-Pentanol	880 [a]	0.70	0.71	1.01	1.05	1.54	0.89	0.72	0.93	1.21
6		1-Hexanol	0.2 [a]	3.52	3.33	3.34	4.42	4.21	3.77	3.59	4.04	4.69
7		1-Octen-3-ol	0.002 [a]	1.39	1.85	2.08	1.94	2.95	2.25	0.96	1.34	1.98
8		1-Heptanol	0.2 [a]	0.23	0.23	0.45	0.36	0.43	0.35	0.24	0.30	0.41
9		4-Thujanol	NA	0.31	0.32	0.58	0.30	0.50	0.45	0.18	0.21	0.48
10		(*E*)-2-Octen-1-ol	0.05 [a]	0.17	0.22	0.38	0.21	0.38	0.34	0.22	0.20	0.00
11		2-ethyl-1-Hexanol	0.27 [b]	0.32	0.32	0.32	0.48	0.17	0.57	0.23	0.29	0.74
12		4-Ethylcyclohexanol	NA	0.14	0.21	0.53	0.16	0.45	0.38	0.07	0.16	0.33
13		Linalool	0.5 [a]	1.55	1.46	2.00	1.51	1.62	1.61	1.16	1.11	1.66
14		1-Octanol	0.054 [a]	0.46	0.41	0.58	0.56	0.68	0.49	0.44	0.43	0.61
15		Terpinen-4-ol	NA	1.49	1.42	1.41	1.32	1.73	1.53	1.12	0.98	1.71
16		α-Terpineol	NA	1.04	0.92	1.09	1.06	1.31	1.05	0.78	0.64	1.06
17		Benzyl alcohol	900 [a]	0.00	0.07	0.09	0.08	0.10	0.08	0.06	0.00	0.11
18		Phenylethyl Alcohol	0.14 [b]	1.18	1.06	0.94	1.14	1.29	0.70	0.65	0.66	1.60
19	Ethers	Eucalyptol	0.23 [b]	7.64	7.61	7.11	8.64	7.61	7.44	6.20	6.37	8.91
20		Linalool oxide	0.1 [a]	0.10	0.00	0.12	0.11	0.23	0.10	0.00	0.00	0.12
21	Aldehydes	Acetaldehyde	NA	0.57	0.00	1.06	0.50	0.83	0.00	0.76	0.60	0.99

22		2-methyl-Butanal	NA	0.00	0.18	0.53	0.09	0.50	0.61	0.00	0.15	0.76
23		3-methyl-Butanal	NA	0.16	0.54	1.35	0.33	0.89	1.69	0.14	0.52	1.75
24		Pentanal	0.2 [a]	0.65	0.73	0.85	1.03	1.29	1.49	0.59	0.86	1.07
25		Hexanal	0.21 [a]	4.47	4.57	4.62	6.85	6.94	7.29	5.14	6.51	7.03
26		Heptanal	0.05 [a]	0.74	0.76	1.05	1.01	1.21	1.41	0.81	0.93	1.25
27		Octanal	0.0001 [a]	0.44	0.63	0.87	0.50	0.52	0.63	0.46	0.38	0.54
28		(*E*)-2-Heptenal	0.051 [b]	0.28	0.32	0.56	0.29	0.57	0.79	0.11	0.14	0.30
29		Nonanal	0.13 [a]	3.15	3.39	3.57	3.97	6.30	6.55	2.37	4.16	6.08
30		5-Ethylcyclopent-1-enecarboxaldehyde	NA	0.11	0.16	0.24	0.13	0.23	0.17	0.07	0.10	0.16
31		Benzaldehyde	0.3 [a]	1.26	1.14	1.53	1.29	1.59	1.63	0.97	1.37	1.71
32		Benzeneacetaldehyde	0.004 [b]	0.22	0.00	1.05	0.37	0.58	0.83	0.00	0.31	0.95
33		(*E*, *E*)-2,4-Decadienal	0.0005 [a]	0.06	0.06	0.49	0.00	0.22	0.16	0.00	0.00	0.00
34		(*E*)-3,7-dimethyl-2,6-octadienal	0.03 [b]	0.00	0.00	0.00	0.00	0.00	0.00	0.14	0.09	0.16
35	Ketones	Acetoin	NA	0.32	0.35	0.00	0.49	0.58	0.00	0.00	0.43	0.41
36		(*E*)-6,10-dimethyl-5,9-undecadien-2-one	NA	0.14	0.08	0.00	0.08	0.15	0.00	0.00	0.00	0.00
37		2-Butanone	0.014 [b]	0.28	0.37	0.22	0.39	0.44	0.28	0.26	0.39	0.31
38		2-Heptanone	0.68 [a]	0.00	0.13	0.21	0.17	0.21	0.22	0.00	0.16	0.21
39		2-Octanone	0.05 [a]	0.54	0.51	0.62	0.59	0.57	0.53	0.55	0.48	0.41
40		6-methyl-5-Hepten-2-one	0.05 [b]	0.28	0.34	0.39	0.36	0.00	0.32	0.46	0.53	0.51
41		Piperitone	NA	0.38	0.37	0.39	0.38	0.46	0.37	0.27	0.21	0.18
42		2,3-Pentanedione	0.005 [a]	0.00	0.00	0.00	0.00	0.13	0.13	0.00	0.00	0.00
43		2,3-Butanedione	0.01 [a]	0.00	0.00	0.00	0.00	0.00	0.35	0.00	0.00	0.00
44	Esters	n-Caproic acid vinyl ester	NA	0.43	0.49	0.48	0.51	1.01	0.70	0.79	0.38	0.27

45		Linalyl acetate	NA	0.68	0.74	0.58	0.73	0.43	0.45	0.83	0.55	0.41
46		Dimethyl phthalate	NA	0.08	0.06	0.07	0.11	0.00	0.00	0.00	0.00	0.00
47		Phenol, 2-methoxy-4-(2-propenyl)-, acetate	NA	0.15	0.08	0.14	0.19	0.15	0.12	0.08	0.10	0.16
48	Hydrocarbons	Tridecane	NA	0.38	0.33	0.00	0.41	0.00	0.27	0.54	0.32	
49		Octane	0.94 [a]	0.43	0.50	0.88	0.75	1.13	0.74	0.95	0.73	0.38
50		1,1,3-trimethyl-Cyclohexane	NA	0.24	0.00	0.34	0.00	0.53	0.30	0.44	0.24	0.43
51		Nonane	NA	0.27	0.25	0.30	0.45	0.32	0.34	0.36	0.20	0.00
52		Decane	NA	0.76	0.59	0.81	0.88	0.81	0.86	0.74	0.78	0.18
53		Undecane	NA	0.84	0.71	0.81	1.10	1.14	1.18	0.95	1.01	0.70
54		Dodecane	NA	1.80	1.22	1.81	1.76	1.36	1.65	1.42	1.24	0.68
55		Pentadecane	NA	0.64	0.68	0.65	0.80	0.71	0.00	0.00	0.00	0.00
56		Caryophyllene	0.064 [b]	1.19	0.00	0.00	0.97	0.00	0.00	1.58	1.28	0.55
57		3-Thujene	NA	0.75	0.69	0.65	0.72	0.68	0.65	0.82	0.54	0.00
58		β-Myrcene	0.1	1.28	1.26	1.06	0.82	0.86	0.91	1.13	0.96	0.61
59		α-Terpinene	NA	0.89	0.87	0.81	0.84	0.79	0.77	0.89	0.61	1.47
60		D-Limonene	0.2 [b]	7.24	7.31	6.01	5.89	4.46	3.88	6.14	4.32	0.70
61		β-Thujene	NA	1.00	0.78	0.68	0.76	0.60	0.64	1.49	0.99	3.91
62		γ-Terpinene	NA	1.32	1.24	1.22	1.23	1.07	1.05	1.26	0.85	1.07
63		Toluene	0.14 [a]	0.66	0.61	0.56	0.75	0.61	0.72	0.65	0.92	1.16
64		Ethylbenzene	3 [a]	0.25	0.23	0.23	0.33	0.31	0.27	0.27	0.30	0.46
65		p-Xylene	NA	0.38	0.33	0.36	0.49	0.39	0.42	0.42	0.41	0.72
66		1,3-dimethyl-Benzene	NA	0.81	1.03	0.71	1.02	1.02	1.03	1.47	0.93	1.60
67		o-Xylene	NA	0.33	0.45	0.33	0.64	0.60	0.67	0.36	0.41	1.01

68		o-Cymene	NA	0.98	1.01	0.88	0.95	0.75	0.74	0.93	0.67	0.97
69	Heterocyclic Compounds	2-pentyl-Furan	NA	0.60	0.78	1.41	0.83	1.20	1.24	0.67	0.88	1.47
70		1-(2-furanyl)-Ethanone	NA	0.00	0.07	0.45	0.00	0.09	0.52	0.00	0.00	0.53
71		3-Furaldehyde	NA	0.10	0.22	0.85	0.15	0.32	0.92	0.10	0.22	1.11
72		Pyrazine	NA	0.00	0.13	0.54	0.00	0.16	0.68	0.00	0.15	0.68
73		methyl-Pyrazine	NA	0.17	0.51	1.98	0.29	0.74	2.61	0.20	0.53	3.03
74		ethyl-Pyrazine	NA	0.00	0.00	0.75	0.00	0.00	0.78	0.00	0.00	1.05
75		2,3-dimethyl-Pyrazine	NA	0.00	0.09	0.51	0.00	0.14	0.62	0.00	0.10	0.68
76		trimethyl-Pyrazine	NA	0.00	0.07	0.30	0.00	0.16	0.53	0.00	0.00	0.52
77		Pyrazinamide	NA	0.00	0.12	0.43	0.00	0.16	0.69	0.00	0.15	0.61
78		2,4,6-trimethyl-Pyridine	NA	0.87	0.86	0.85	0.81	0.77	0.94	1.18	0.80	0.78
79		3-Furanmethanol	NA	0.24	0.52	2.45	0.29	0.94	2.85	0.21	0.61	3.85
80	Phenols	Eugenol	0.09 [b]	27.50	14.51	23.70	27.35	28.72	20.12	14.15	17.12	24.69
81		2,4-Di-tert-butylphenol	NA	0.29	0.63	0.67	0.40	0.47	0.48	0.43	0.47	0.38
82		trans-Isoeugenol	NA	0.71	0.41	0.67	0.92	0.72	0.61	0.44	0.53	0.77
83		p-Cresol	0.1 [b]	0.31	0.24	0.30	0.31	0.35	0.30	0.09	0.26	0.45
84	others	Anethole	0.1 [a]	2.05	2.21	1.12	1.93	1.18	1.14	1.22	1.50	2.07
85		Butylated Hydroxytoluene	NA	8.20	5.44	6.43	7.83	6.91	5.93	5.39	4.96	9.37
86		Maltol	NA	0.00	0.00	0.55	0.00	0.00	0.60	0.00	0.00	0.51
87		Methanethiol	0.002 [a]	0.00	0.00	0.00	0.87	0.41	0.00	0.19	0.30	0.00

[a] The thresholds for some compounds were obtained from *Compilations of Odor Threshold Values in Air, Water, and Other Media* (2nd enlarged and revised edition). [b] The thresholds for some compounds were derived from the literature [58–61] and the Flavor Database of Shanghai Jiao Tong University (https://mffi.sjtu.edu.cn/database).
